# Association of Socioeconomic Status and Overweight/Obesity in Rural-to-Urban Migrants: Different Effects by Age at Arrival

**DOI:** 10.3389/fpubh.2020.622941

**Published:** 2020-12-17

**Authors:** Ye Wang, Li Pan, Shaoping Wan, Huowuli Yi, Fang Yang, Huijing He, Zheng Li, Zhengping Yong, Guangliang Shan

**Affiliations:** ^1^Department of Epidemiology and Statistics, School of Basic Medicine, Institute of Basic Medical Sciences, Chinese Academy of Medical Sciences, Peking Union Medical College, Beijing, China; ^2^Sichuan Cancer Center, School of Medicine, Sichuan Cancer Hospital and Institute, University of Electronic Science and Technology of China, Chengdu, China; ^3^Puge Center for Disease Control and Prevention, Liangshan, China; ^4^Xichang Center for Disease Control and Prevention, Liangshan, China; ^5^Sichuan Academy of Medical Sciences and Sichuan Provincial People's Hospital, Chengdu, China

**Keywords:** overweight, obesity, socioeconomic, China, Yi migrant

## Abstract

This study aims to investigate the association between socioeconomic status and overweight/obesity in rural-to-urban Yi migrants in China, and to explore whether the association varied by the age at arriving urban areas. The cross-sectional population-based data from the Yi Migrants Study in 2015 was used, which included 1,181 Yi migrants aged 20–80 years. Socioeconomic status was evaluated by education level, personal annual income, and a composited variable (socioeconomic status index, SESI). Measured weight and height were used to calculate BMI and to define overweight/obesity (BMI ≥ 24 kg/m^2^). The results suggested that the association of income and SESI with overweight/obesity was not significant when age at arrival (two groups, <20 and ≥20 years) was considered as a covariate. In the stratification analysis, reversed association was observed in the two groups of age at arrival. In migrants of <20 years of age at arrival, higher level of education and SESI were related to decreased risk of overweight/obesity. In contrary, in those of ≥20 years at arrival, higher socioeconomic status level was found to be related to increased risk. Our findings suggest that the effect of socioeconomic status on overweight/obesity was modified by the age at arrival in Yi migrants. Especially, the association between socioeconomic status and overweight/obesity was negative when migration before 20 years of age, and transfer to positive after 20 years.

## Introduction

Obesity is a fast-growing public health problem that has raised worldwide concern. The prevalence of overweight and obesity in the past decades has reached epidemic proportions in many countries (1). Obesity is not only defined as a chronic disease, but also substantially increases the risk of metabolic diseases, cardiovascular diseases, and some types of cancer (2). The World health organization (WHO) defines obesity as excessive fat accumulation that might impair health. It is recognized that obesity is the result of the interplay between comprehensive factors including genetic and environmental determinants (3).

Socioeconomic status (SES) has been identified as one of the factors that related to obesity. But recent updates demonstrated inconsistent associations across populations. Some evidences from developed countries revealed that low-SES groups were at increased risk of obesity than their high-SES counterparts (4, 5). While in low- or middle-income countries, the association became mixed, showing a variation from positive to none or inverse relationship (6, 7). Even in China, disparities in the relationship was found across regions, gender, and rural/urban areas (8, 9).

Urbanization in China is rapidly proceeding and the scale of rural-to-urban migration is gradually expanding (10). The transition in socioeconomic status and lifestyle patterns in rural-to-urban migrants may lead to increase in risk of obesity, and migration studies provide valuables information on the effects of environment factors transition on health. Yi people is the population settled in isolated mountainous areas in southwest China, and was known as low prevalence of hypertension and obesity. Since the 1950s, some Yi farmers migrated to urban areas for living and working. Previous work of the Yi Migrant Study has well-demonstrated the effects of rural-to-urban migration on chronic diseases (11–13). But the relationship between SES and obesity in Yi migrants is not clear, especially existing the fact that the SES varied by acculturation groups in migrants. Using cross-sectional data from the Yi Migrants Study in 2015, this study investigated the association between SES and overweight/obesity in rural-to-urban Yi migrants, and to explore whether the association varied by the age at arrival.

## Methods

### Study Population

A cross-sectional survey was conducted in April and November 2015 in Liangshan Yi Autonomous Prefecture, Sichuan province, China. The Yi Migrant Study was designed to assess cardiovascular risk factors in rural-to-urban Yi migrants. Details of the sampling procedures have been described previously (14). Briefly, a stratified cluster sampling method was used to select participants aged 20–80 years. In the first stage, the autonomous prefecture capital, Xichang city, and an economic backward area, Puge county were selected. In the second stage, three urban districts in Xichang city and the town with county administration seats in Puge county were selected to enroll Yi migrants. In the final stage, residents in the selected areas were all invited to participate in the survey. In this study, Yi people were identified as individuals whose parents were both of Yi ethnicity. Yi migrants were defined to be Yi people who were born in rural areas and then migrated to urban areas for at least 1 year. All participants provided written informed consent before the survey.

### Measurements

Demographic information, socioeconomic data and lifestyle factors were collected by face to face interviews. The interview was conducted by well-trained medical staff using standard questionnaire. Body height and weight were measured with bared feet and light clothing using fixed stadiometer and body composition analyzer (BC-420, TANITA, Japan), with the accuracy to 0.1 cm and 0.1 kg, respectively. The average of two height measurements was recorded.

### Dependent Variable

The continuous outcome, body mass index (BMI) was calculated as the weight in kilograms divided by the square of height in meters. The binary variable, overweight/obesity was defined as BMI ≥ 24 kg/m^2^, according to the recommendation suitable for Chinese population (15).

### Independent Variables

The acculturation of Yi migrants was measured through age at arrival and length of residence in urban areas, which represent the extent of exposure to the urban environment. By asking respondents the year and month they first moved from rural to urban areas, the age of arrival and length of residence were calculated according to the birth date, move date, and survey date. Under the consideration of sample size, sample characteristics, and round-off number, age at arrival of the subjects was classified into two groups: <20 and ≥20 years, and length of residence was classified as 1–9, 10–19, and ≥20 years.

Socioeconomic status was evaluated by two direct variables: education level and personal annual income, and one compositive variable: socioeconomic status index (SESI). Education was categorized into three categories based on the participants' highest educational attainment: illiterate, primary or middle school (equivalently <12 years of education), and high school or above (equivalently ≥12 years of education). Personal annual income was categorized into two groups in accordance with the round-off number of the median (<20,000 and ≥20,000 CNY/year). SESI was composited by assigning a score to education and income. The assignment was 0 point for illiterate and <20,000 CNY/year, 1 point for primary or middle school and ≥20,000 CNY/year, and 2 points for high school or above. Combining the score for education and income, SESI was categorized as low (0 point), middle (1 point), and high (2 or 3 points).

Other covariates included were smoking, alcohol drinking, occupational physical activity, and leisure-time exercise. Both smoking and drinking were grouped into current, former, and never. Occupational physical activity was grouped into light, moderate, and heavy according to the intensity of daily work. Leisure-time exercise was considered as participation in moderate or vigorous activity for at least 20 min at leisure time, with three levels as light (<1 day per week), moderate (1–4 days per week), and heavy (5–7 days per week).

### Statistical Analyses

Descriptive statistics was performed stratified by age at arrival and length of residence. Chi-square test and variance analysis were used for categorical and continuous variables, respectively.

To examine whether there was significant association between socioeconomic status with outcomes, the following modeling strategies were applied. Initially, a linear regression model with BMI as the outcome and socioeconomic variables as the primary interested variable, while controlling for covariates including age at arrival. As SESI was derived from education and income, two separated models were fitted: (a) model included education and income, and (b) model only included SESI. Second, logistic regression models with overweight/obesity as the outcome were used to estimate odds ratio (OR) and 95% confidence interval (CI) following the above modeling rules. Due to the complex survey design, logistics regression models used took account of the complexity of the stratified cluster sampling strategy (16).

Thereafter, an interaction term was included in the logistic models to test the hypotheses that the association between socioeconomic status and overweight/obesity may be modified by the age at arrival. These three socioeconomic indicators were evaluated independently of each other by including one interaction term at a time. Finally, to determine how the association was differed, logistic regression models were fitted with stratification by age at arrival. We also test the interaction between length of residence and socioeconomic status, but no significant interaction term was found, thus we did no further analysis. All regression analyses were controlled for the following covariates: age (starting at 20 and grouped per 10 years), gender, smoking, drinking, physical activity, exercise, and length of residence.

A two-tailed *p*-value of <0.05 was considered statistically significant. All analyses were conducted using SAS statistical software (Version 9.4; SAS Institute Inc., Cary, NC, USA).

## Results

Table 1 lists the characteristics of the study sample of by age at arrival and length of residence. This study included 33.11% males and 66.89% females, with the mean age at 48.90 ± 14.42 years. Between the two groups of age at arrival, there was significant difference in demographic characteristics. Compared to respondents who were <20 years at arrival, migrants of ≥20 years at arrival had older age, were less likely to drink, and less likely to participant in leisure-time exercise. The mean BMI of the two groups was not significantly different, while the crude prevalence of overweight/obesity was higher in ≥20 years at arrival than that in their counterparts. Among the groups of length of residence, respondents with ≥20 years of residence had the oldest age, the most proportion of current smoking, current drinking, light occupational physical activity, and heavy leisure-time exercise. The mean BMI and crude prevalence of overweight/obesity increased considerably with the duration of residence in urban areas.

**Table 1:** Characteristics of the study sample by age at arrival and length of residence.

	**Total (*****n*** **=** **1,181)**		**Age at arrival (years)**					**Length of residence (years)**						
			** <20 (*****n*** **=** **378)**		**≥20 (*****n*** **=** **803)**		***P***	**1–9 (*****n*** **=** **343)**		**10–19 (*****n*** **=** **337)**		**≥20 (*****n*** **=** **501)**		***P***
	**No**.	**%**	**No**.	**%**	**No**.	**%**		**No**.	**%**	**No**.	**%**	**No**.	**%**	
Gender							0.006							<0.001
Male	391	33.11	146	38.62	245	30.51		97	28.28	92	27.30	202	40.32	
Female	790	66.89	232	61.38	558	69.49		246	71.72	245	72.70	299	59.68	
Mean age (years)	48.90	(14.42)	43.60	(16.33)	51.39	(12.69)	<0.001	44.73	(14.48)	44.01	(13.67)	55.03	(12.49)	<0.001
Age group (years)							<0.001							<0.001
20–	100	8.47	79	20.90	21	2.62		52	15.16	45	13.35	3	0.60	
30–	260	22.02	116	30.69	144	17.93		86	25.07	106	31.45	68	13.57	
40–	274	23.20	58	15.34	216	26.90		91	26.53	80	23.74	103	20.56	
50–	213	18.04	44	11.64	169	21.05		42	12.24	53	15.73	118	23.55	
60–	240	20.32	48	12.70	192	23.91		57	16.62	34	10.09	149	29.74	
70–80	94	7.96	33	8.73	61	7.60		15	4.37	19	5.64	60	11.98	
Smoking							0.475							<0.001
Never	806	68.25	249	65.87	557	69.36		246	71.72	246	73.00	314	62.67	
Former	66	5.59	22	5.82	44	5.48		8	2.33	15	4.45	43	8.58	
Current	309	26.16	107	28.31	202	25.16		89	25.95	76	22.55	144	28.74	
Drinking							<0.001							<0.001
Never	750	63.50	202	53.44	548	68.24		244	71.13	222	65.87	284	56.69	
Former	89	7.54	29	7.67	60	7.47		18	5.25	22	6.53	49	9.78	
Current	342	28.96	147	38.89	195	24.28		81	23.62	93	27.60	168	33.53	
Occupational physical activity							0.503							<0.001
Light	908	76.88	295	78.04	613	76.34		242	70.55	238	70.62	428	85.43	
Moderate	179	15.16	58	15.34	121	15.07		68	19.83	68	20.18	43	8.58	
Heavy	94	7.96	25	6.61	69	8.59		33	9.62	31	9.20	30	5.99	
Leisure-time exercise							0.002							<0.001
Light	525	44.53	143	37.93	382	47.63		201	58.60	181	53.87	143	28.60	
Moderate	274	23.24	89	23.61	185	23.07		74	21.57	87	25.89	113	22.60	
Heavy	380	32.23	145	38.46	235	29.30		68	19.83	68	20.24	244	48.80	
Mean BMI (kg/m^2^)	24.47	(3.81)	24.27	(3.68)	24.56	(3.87)	0.232	23.74	(3.74)	24.29	(3.87)	25.08	(3.73)	<0.001
Overweight/obesity	625	52.92	183	48.41	442	55.04	0.038	156	45.48	173	51.34	296	59.08	<0.001

*Numbers in parentheses are standard deviation*.

**Figure 1:**
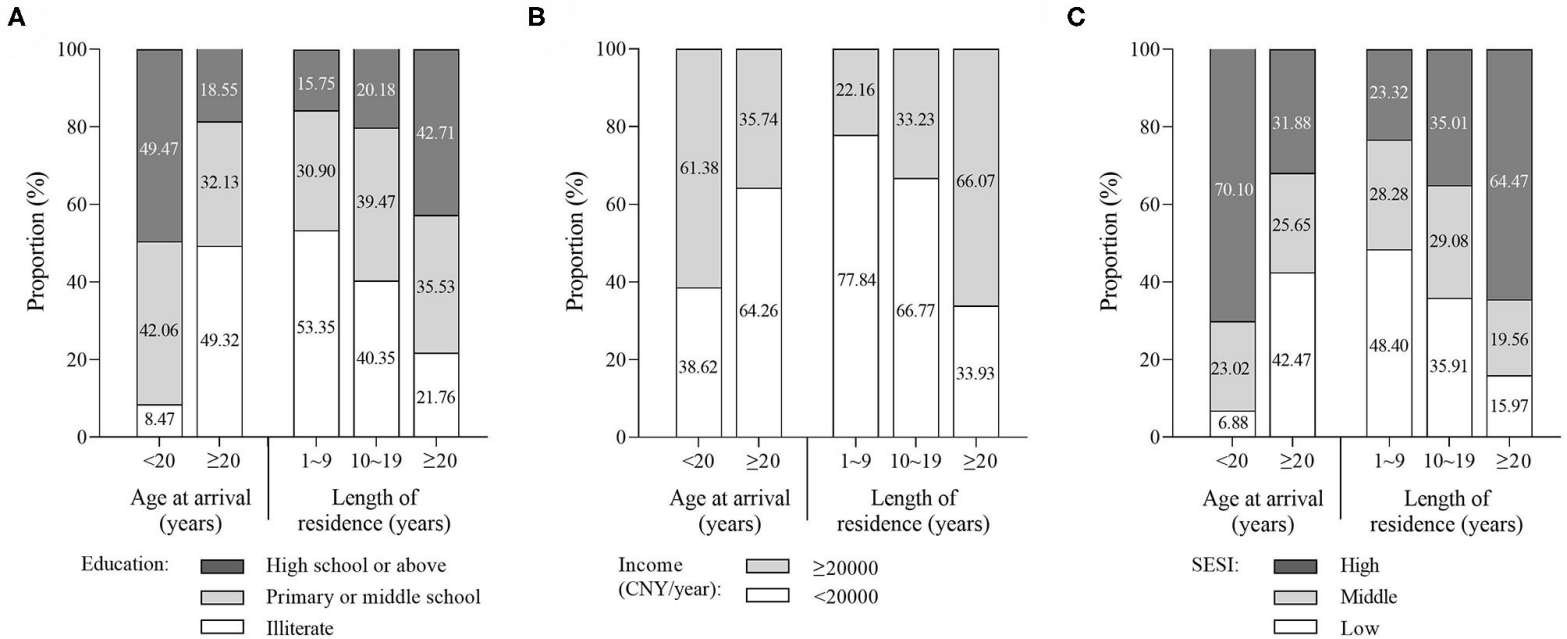
Disparities in education **(A)**, income **(B)**, and socioeconomic status index (SESI) **(C)** by age at arrival and length of residence.

Bar chart (Figure 1) clearly shows the disparities in socioeconomic status of Yi migrants with different age at arrival and length of residence. Compared to their counterparts of ≥20 years at arrival, migrants of <20 years at arrival were better educated, had higher income, and had a higher proportion of high SESI level. Of the migrants with <20 years at arrival, nearly half had the high school education level or above, while in respondents with ≥20 years of arrival, nearly half of them were uneducated. In migrants of <20 years at arrival, the proportion of high-income level (≥20,000 CNY/year) was 61.38%, corresponding 35.74% in migrants of ≥20 years at arrival. Combining education and income, most respondents with <20 years of arrival had high SESI level, and only 6.88% of them had low SESI level. The distribution of SESI in migrants of ≥20 years at arrival was relative balanced. There was an apparent tendency that the distribution of socioeconomic status changed with length of residence. The proportions of high education level, high income, as well as high SESI level raised with the length of residence increased.

Table 2 reports the estimated coefficients and odds ratios for the multiple regression models assessing the relationship between socioeconomic status and outcomes. These models used the full sample, which means the age at arrival was include as a covariate. The results reveal that after adjusting for potential confounding factors including age, gender, age at arrival, length of residence, etc., significant association was only found between education with overweight/obesity.

**Table 2:** Effects of socioeconomic status on BMI and overweight/obesity by multiple regression models.

	BMI^*^		Overweight/obesity^**^	
	**β**	**95% CI**	**OR**	**95% CI**
**Model 1**				
**Education**				
Illiterate	0.00 (ref)	–	1.00 (ref)	–
Primary or middle school	−0.10	−0.68, 0.49	0.86	0.76, 0.98
High school or above	0.16	−0.60, 0.91	1.12	1.00, 1.26
**Income (CNY/year)**
<20,000	0.00 (ref)	–	1.00 (ref)	–
≥20,000	0.17	−0.38, 0.71	1.06	0.88, 1.28
**Model 2**				
**SESI**				
Low	0.00 (ref)	–	1.00 (ref)	–
Middle	−0.10	−0.71, 0.51	0.91	0.73, 1.15
High	0.14	−0.50, 0.78	1.04	0.90, 1.21

* *General linear regression models were applied*.

** Logistic regression models were applied.

*Models adjusted for age, gender, smoking, drinking, occupational physical activity, leisure-time exercise, age at arrival, and length of residence*.

**Figure 2:**
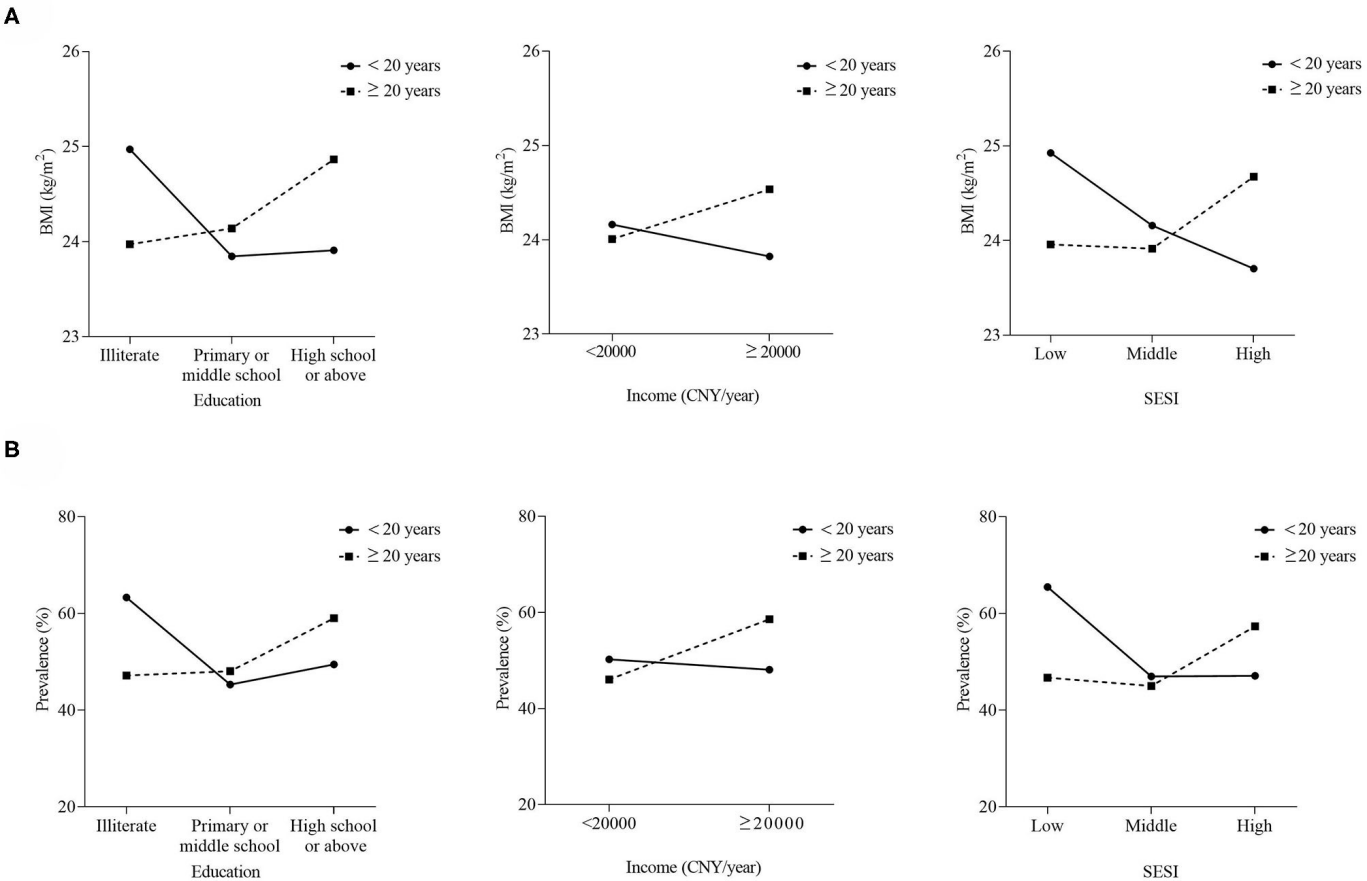
Trends of BMI **(A)** and overweight/obesity prevalence **(B)** with socioeconomic status by age at arrival. BMI was adjusted for age, gender, smoking, drinking, occupational physical activity, leisure-time exercise, and length of residence; prevalence was age and sex standardized.

As significant interaction terms were detected between SES and age at arrival (*P* < 0.0001 for education, *P* = 0.0007 for income, and *P* < 0.0001 for SESI, respectively), the sample was stratified by age at arrival to show different patterns of BMI and overweight/obesity between groups. Figure 2 graphs how the effects of SES on outcomes varied by age at arrival. Figure 2A shows that the trends of adjusted BMI with socioeconomic status were opposite in the two age at arrival groups. In migrants of <20 years at arrival, the highest BMI presented to the illiterate, <20,000 CNY/year, and low SESI groups. Conversely, in migrants of ≥20 years at arrival, the trends between socioeconomic status and BMI shifted from negative to positive. The highest BMI presented to the high school or above, ≥20,000 CNY/year, and high SESI groups. Similar patterns of overweight/obesity prevalence between the two groups were also displayed in Figure 2B.

**Figure 3:**
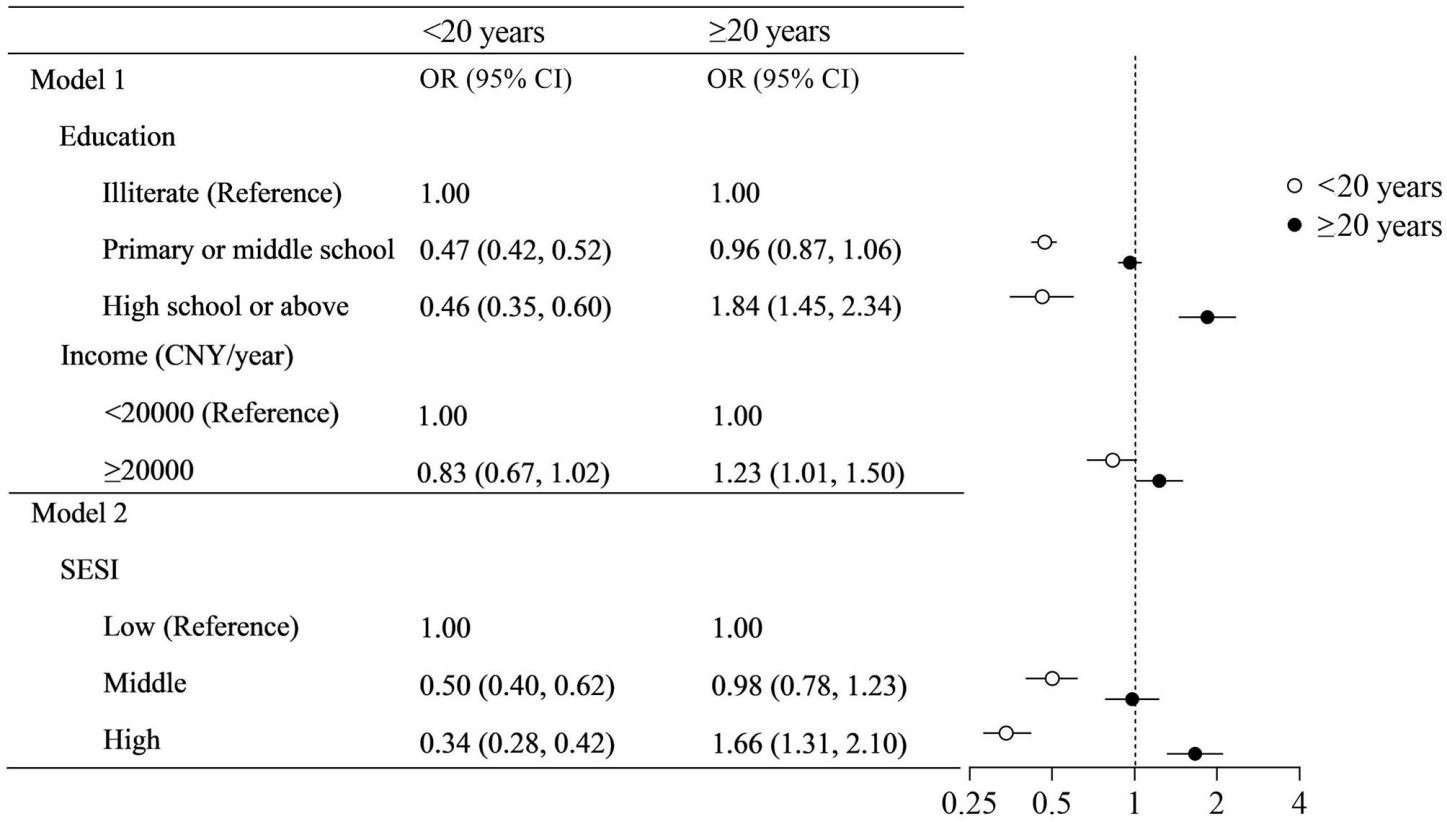
Association of socioeconomic status with overweight/obesity by age at arrival. Models adjusted for age, gender, smoking, drinking, occupational physical activity, leisure-time exercise, and length of residence.

Forest plot (Figure 3) shows the association between socioeconomic status and overweight/obesity in stratification analysis. In migrants of <20 years at arrival, there was a negative association of education and SESI with overweight/obesity. Compared to illiterate, odds ratios for middle and high education level were 0.47 (0.42–0.52) and 0.46 (0.35–0.60). Compared to low SESI level, odds ratios for middle and high SESI groups were 0.50 (0.40–0.62) and 0.34 (95% CI: 0.14–0.86). In contrast, in migrants of ≥20 years at arrival, higher SES level was positively related to overweight/obesity risk. Compared to illiterate group, odds ratio for high school and above was 1.84 (95% CI: 1.45–2.34). The odds ratio for higher income group was 1.23 (1.01–1.50) compared to lower income group. The odds ratio for high SESI level was 1.66 (95% CI: 1.31–2.10) relative to low SESI level.

## Discussion

In this population-based cross-sectional study, socioeconomic status was found as an independent determinant of overweight/obesity in Yi rural-to-urban migrants in China. The results showed that the relationship of SES with overweight/obesity in Yi rural-to-urban migrants was modified by age at arrival, which was found out that without stratification, the association was not significant especially for income and SESI. This study revealed that in Yi migrants of <20 years at arrival, SES was negatively associated with overweight/obesity, controlling for age, gender, smoking, drinking, occupational physical activity, leisure-time exercise, and length of residence. On the contrary, in those of ≥20 years at arrival, higher SES groups were related to increased risk.

As a widely recognized risk factor, socioeconomic status and its relationship with obesity has been explored in numerous studies (17–20). The indicators used to reflect SES as well as their classifications in these studies varied, and the mainly used variables were education, income, and occupation (7–9). In the present study, two of these conventional indicators were selected (education and income). We did not include occupation because the age range of the participants was wide (20–80 years), which means the older part of the study population were mostly retired or unemployed. Besides, there is a correlation among the SES indicators, and the variation in occupation can be largely explained by education and income (21, 22). As to income, to some extent, it can reflect the accessibility to food and health care services, and the fact is, part of rural-to-urban migrants especially the elderly was not engaged in a stable work to earn a monthly income. So, we conducted a conservative classification by the median value. As opposed to income and occupation, education is acquired in early life stage and extending throughout lifetime. Among these SES indicators, education is considered as the most stable component to reflect health awareness, health care ability, and lifestyle patterns, etc. (22, 23). In this study, a new comprehensive indicator (socioeconomic status index, SESI) was composited by education and income, in which education accounted for a heavier weight (24). The results suggested that SESI was a stable variable and did not altered the relationship of SES with overweight/obesity in the study population.

Previous studies concluded that the relationship between SES and obesity differed by the country's level of development, which means that positive associations between SES and obesity were more common in less developed countries, while inversed association were commonly found in developed countries (25). Following the modernization and dramatic economic development over the past decades, China has experienced a transition from the history of undernutrition to a rapid increase in obesity (26). The patterns of SES-obesity relationship can be traced across the different stage of economic development. A study explored the discernible patterns of obesity epidemic over time and grouped the obesity transition into conceptual stages (27), among which China is experiencing the stage characterized by narrowing gap or a reversal of socioeconomic differences in obesity. A study from the urban and suburban region in north China showed that higher monthly income and education were related to decreased odds of abdominal overweight/obesity (9). Another study focusing on the rural residents in northwest China revealed that in men, the risk of overweight/obesity and abdominal obesity were higher in the high SES groups, but women with a high level of education were less likely to have overweight/obesity than those with a low level of education (8).

The pathways explaining the relationship of SES and obesity are multiple, including increased energy and fat consumption (28, 29) and decreased density of physical activity (30–32). A systematic review which evaluated the behavioral risk factors in low-income and lower-middle-income countries found that behaviors, such as alcohol drinking, tobacco use, physical activity, and food consumption (e.g., fruit, fats, processed food, etc.) were at difference between low and high SES groups (30). The novel findings in this study are notable for several reasons. First, to our knowledge, evaluations on the association of SES and obesity in Chinese rural-to-urban migrants are limited. Second, this study showed that there were significant disparities on SES by age at arrival and length of residence in migrants. Finally, this study stratified the rural-to-urban migrants and found opposed association of SES and overweight/obesity by age at arrival. One of the main points to explain the differed association by age at arrival is educational attainment (33). In their early life stage, the two Yi migrant groups were educated in different environment. The rural-urban disparities in education quality may lead to the differences in health literacy and the accessibility to health information (34). Of those ≥20 years at arrival, with the SES level raising, they may be more likely to change their dietary habit and increase the high-fat energy-dense food intake. On the contrary, those moving from rural to urban areas in childhood were more adapted to the urbanized lifestyle, reflecting in the adherence to healthier behavior factors like leisure time physical activity.

One major limitation of this study is lack of the collection on dietary data. Obtaining reliable and accuracy dietary information in a large-scale population-based field survey is a big challenge, this part was not included in the study. As the socioeconomic is closely related to dietary habit, the bias due to diet cannot be adjusted. In the future, details such as food preference are expected in field work.

In conclusion, the results suggested that the SES-obesity relationship was modified by age at arrival in rural-to-urban Yi migrants. Positive association between SES and overweight/obesity was found in those ≥20 years at arrival, while inversed association was found in those <20 years at arrival. This study calls for the urgent need to improve education attainment especially health literacy. We also emphasize the healthy lifestyle adherence in rural-to-urban migrants.

## Data Availability Statement

The datasets generated or analyzed for this study are available from the corresponding author on reasonable request.

## Ethics Statement

The studies involving human participants were reviewed and approved by the institutional review board of Institute of Basic Medical Sciences, Chinese Academy of Medical Sciences. The patients/participants provided their written informed consent to participate in this study.

## Author Contributions

GS designed the study and supervised data collection. YW analyzed the data, interpreted results, and drafted the manuscript. LP, SW, HY, FY, ZL, and ZY participated in data collection. HH critically reviewed the manuscript. All authors have approved the submitted versions.

## Conflict of Interest

The authors declare that the research was conducted in the absence of any commercial or financial relationships that could be construed as a potential conflict of interest.
